# Role of DNA Methylation in the Pathogenesis of Skin Disorders: Mechanisms, Inhibitors of Methylation-Related Enzyme, and Molecular Docking Studies

**DOI:** 10.1155/bmri/7002918

**Published:** 2025-03-27

**Authors:** Anchal Rani, Gagandeep Kaur, Ravinder Kumar, Rohan Samir Kumar Sachan, Mukesh Kumar, Harsh Sable, Abdel Rahman Mohammad Al Tawaha, Shahid Malik, Arun Karnwal, Tabarak Malik

**Affiliations:** ^1^School of Allied and Health Care Sciences, GNA University, Phagwara, India; ^2^School of Pharmacy, GNA University, Phagwara, India; ^3^Research and Development Cell, Lovely Professional University, Phagwara, Punjab, India; ^4^Sharda School of Allied and Healthcare Sciences, Sharda University, Greater Noida, Uttar Pradesh, India; ^5^Department of Biological Sciences, Al Hussein Bin Talal University, Maan, Jordan; ^6^Department of Biochemistry, All India Institute of Medical Sciences, Gorakhpur, India; ^7^Department of Microbiology, Graphic Era (Deemed to be University), Dehradun, Uttarakhand, India; ^8^Department of Biomedical Sciences, Institute of Health, Jimma University, Jimma, Ethiopia

**Keywords:** dermatitis, DNA methylation, DNMT, psoriasis, skin disorders

## Abstract

DNA methylation is an epigenetic mechanism modulating gene expression without altering the genetic sequence and plays a significant role in skin disorders. Methylation patterns on specific genes can lead to either overexpression or suppression, impacting cellular functions critical to skin health. Skin disorders such as atopic dermatitis, eczema, androgenetic alopecia, systemic lupus erythematosus, psoriasis, and systemic sclerosis have been linked to abnormal DNA methylation, which contributes to disease progression through immune dysregulation, barrier dysfunction, and inflammation. The methylation of genes like S100A2 and FCERIG in atopic dermatitis or FLG in eczema illustrates how modifications affect immune pathways and skin integrity. Recent advancements in DNA methylation analysis have enhanced the precision of detecting methylation levels and their influence on gene expression, leading to a deeper understanding of disease mechanisms. Identifying aberrant methylation patterns offers potential biomarkers for early diagnosis and therapeutic targets, especially in autoimmune and inflammatory skin diseases. Further exploration of epigenetic changes could pave the way for innovative treatments, addressing underlying epigenetic disruptions that characterize these conditions.

## 1. Introduction

Skin is the first line of defense and a sensory organ covering the body's complete external surface. Skin involves two layers: the topmost layer is the epidermis, and the other inner layer is the dermis. The epidermis is a stratified squamous keratinized epithelium made of four evident cells, keratinocytes (KCs) being the most frequent among all participating cells. Additionally, tactile Merkel cells, melanin-producing melanocytes, and antigen-presenting Langerhans cells are the less abundant cells, and nonepithelial cells are interspersed in specific locations in the human body. Moving further towards the second skin layer, the dermis, three different mesenchymal sources derive this layer. The neural crest cell originates from the face and neck dermis, whereas the lateral plate mesoderm derives from the limbs and the body wall, and the back derives from the paraxial mesoderm [1].

About 20%–33% of people have skin diseases; in the United Kingdom, around 54% of the population goes through skin diseases annually. Epigenetics stands for alteration in genetic sequences, so the mechanism of action of a gene is transformed without fluctuating DNA sequence. Methylation, acetylation, phosphorylation, ubiquitylation, and sumoylation are the processes involved in the epigenetic mechanism. Epigenetics is a natural phenomenon required for multifarious body functions, but any irregulating stimulus can have adverse effect to the health of *Homo sapiens*.

In 1983, DNA methylation was identified in cancer patients and has been observed in other diseases and health conditions. Methylation in DNA is defined as the addition of methyl groups in cytosine bases, leading to overexpression or silencing of typical gene pairs. Rollin Hotchkiss was the first scientist to introduce the DNA methylation mechanism, which leads to gene alteration such as mutation, deletion, insertion, and translocation, leading to gene modification [2, 3].

A methyl group from S-adenyl methionine (SAM) was transferred to the fifth carbon of a cytosine residue to form 5mC by DNA methyltransferases (DNMTs), which catalyzed DNA methylation. Several DNMTs, notably DNMT3A and DNMT3B, are known as “de novo DNMTs” because they can change the original DNA's methylation pattern. DNA methyltransferase 1 (DNMT1) is required for DNA replication because it transfers the parent DNA strand's DNA methylation pattern to the daughter DNA strand [3].

### 1.1. Methods to Detect Methylation Level in DNA Sequence

Epigenetics refers to inheritance variations in DNA chromosomes without changing the DNA sequence, further regulating gene expression. One of the most extensively studied epigenetic modifications is DNA methylation, which is the covalent modification of cytosine. This modification offered a molecular mechanism by which cells develop and proliferate.

Recent advancements in DNA detection proved their role in tumor detection and cancer-curing. These applications require modifications of profiling approaches. Some of these detection methods are discussed below [4].

#### 1.1.1. Bisulfite Treatment and DNA Methylation Analysis

One of the most widely used and effective methods in bisulfite treatment involves exposing DNA to sodium bisulfite. This process selectively deaminates nonmethylated cytosines without affecting methylated cytosines, converting them into uracil, which is then read as thymine during a subsequent polymerase chain reaction (PCR). The difference between the melting point of various sequences gives the basis of DNA methylation. The former step is to treat DNA with sodium bisulfite and then run a PCR. After this, the cytosine position is detected. The main advantage of this method is that there is no need for sequencing and cloning processes, and it can be used for quantitative determination [5]. The methylation-sensitive single-nucleotide primer extension (Ms-SnuPE) is another DNA detection technique with more advancements. PCR electrophoresis is used, making this method more sensitive for isolation. The C/T ratio can be easily determined using end-product radiation, which then serves as a template for primer synthesis. This method not only identifies the methylation site of a gene but also measures the level of methylation [6, 7]. Meissner introduced the reduced representation bisulfite-sequencing (RRBS) method for methylation analysis. This method is grounded on an Msp I restriction enzyme to digest the genome, followed by a sequence process to achieve methylation data of each single base pair [8].

Research developed a method called methylation-specific denaturing-gradient-gel-electrophoresis (MS-DGGE) to address the challenge of complex base sequences. This technique leverages the melting temperature differences of 5-methylcytosine sequences for denaturation. As methylated and nonmethylated DNA strands have distinct melting points, they separate at various positions within the gel, allowing the measurement of 5-methylcytosine levels [9]. The fluorescence method is also used to determine methylation level; in this method, fluorescence intensity is detected after a fluorescence color labels DNA filaments. Melting points are also determined for methylated and nonmethylated genes since they have different melting points [10, 11].

#### 1.1.2. Biological Identification Method to Determine Methylation Level in DNA

**Figure 1:**
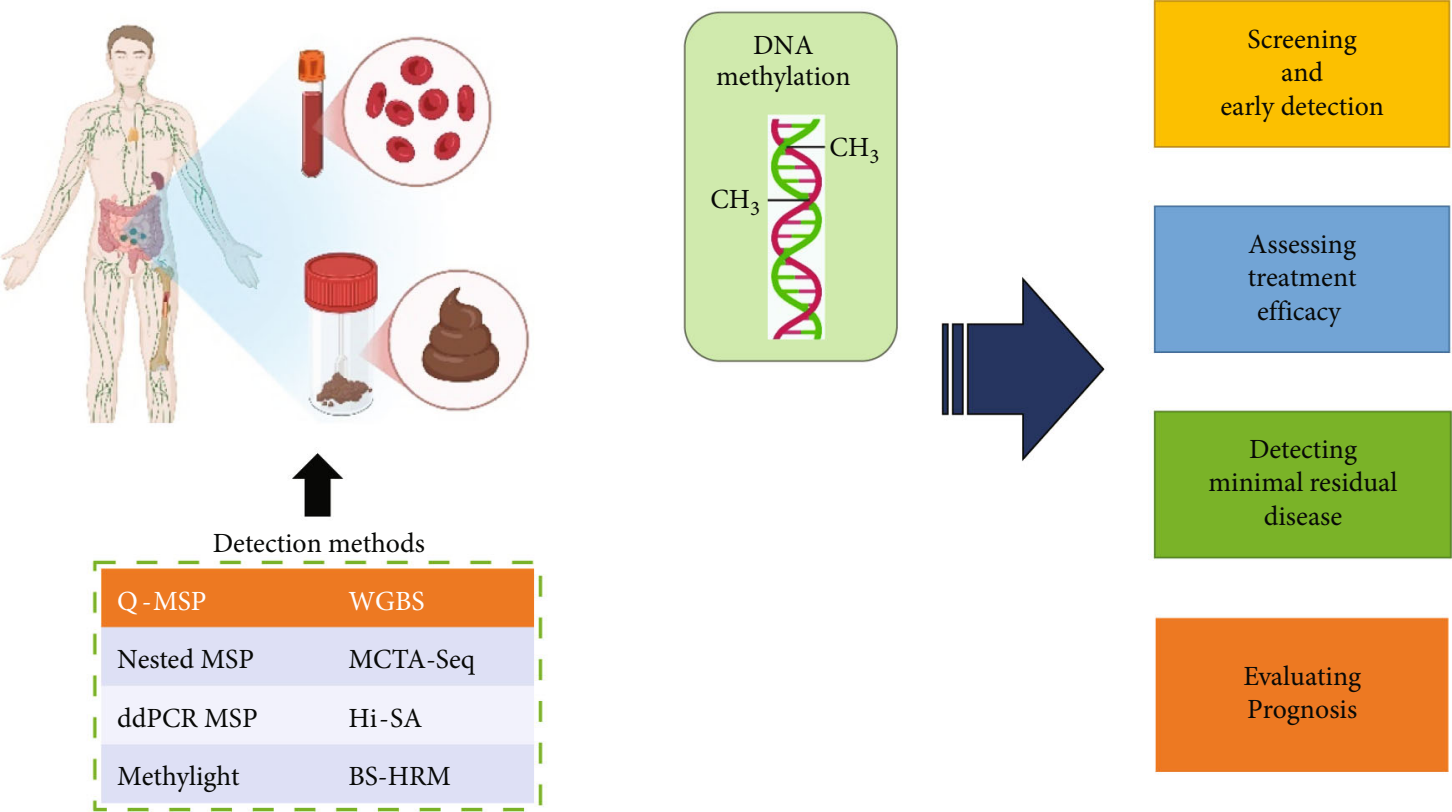
Main technologies for DNA methylation detection and clinical applications.

Utilization of enzymes to detect DNA methylation is also possible as these methods offer more specificity (Figure 1). A pair of isoschizomers and nonisoschizomers is used to cleave the required identical sequences but has different sensitivity to the methylation level. SmaI, Not I, Hpa II, and BstU I are methylation-sensitive restriction enzymes that separate DNA pairs. They only cut nonmethylated DNA and do not influence methylated DNA. The results of this method are based on revealing nonmethylated DNA locations but targeting enzyme sites. The only drawback is that this technique covers small genome levels due to limited CpG sites [4].

The second technique that comes under biological identification is enzyme-related techniques. The restriction-landmark genomic scanning (RLGS) approach is the first example where Not I restriction enzyme digested DNA samples. This digestion will give the idea of methylated sites. Electrophoresis is run after separating the base pair with restriction enzymes, which analyzes various CpG sites' methylation statuses. The limitation of this process is the reliability of the results. An enhanced enzymatic digestion technique that produces more precise results is the methylation-sensitive restriction-endonuclease PCR/southern (MS-RE-PCR) method [12].

This technique uses restriction enzymes Msp I and Hpa II to treat the DNA samples and detect 5-CCGG-3 sequences. From these restriction enzymes, Msp I could not clip nonmethylated cytosine in the DNA sample and only separated methylated C in 5-CmCGG-3. Following this procedure, PCR was used to detect the level of methylated cytosine. Another method utilizes a pair of fluorophores, and an extinguisher is designated beside the molecular beam radius. The fluorophore signals intensify, indicating the concentration of methylation when 5-methylcytosine fragments are generated in the probe by the Dam MTase catalyst. However, the high cost of this process limits its widespread use [13].

Zinc finger proteins (ZF), MBD, and anti-5-methylcytosine IgG1 antibodies are employed in the biodependence reaction to assess the methylation levels in a sample. This method relies on the attachment of methylated DNA sequences to MBD proteins. To detect 5-methylcytosine in the genome, microarray (MBD-chip) or sequencing technologies such as MBDCap-seq and MethylCap-seq are utilized [6].

#### 1.1.3. Bisulfite-Free and Enzyme-Free Techniques

A method for DNA methylation that does not require enzymes or bisulfite is proposed to overcome the limitations of bisulfite conversion techniques and biological methods. This technique involves oxidizing thymine and 5-methylcytosine by osmium tetroxide in a dual C5–C6 bond, which causes pyrimidine to undergo direct electrochemical oxidation to analyze DNA methylation. The multiwalled carbon nanotubes (MWCNTs) supported by a choline chloride monolayer were also employed as an electrochemical technique to detect DNA methylation [14, 15]. The reason behind this is the electrocatalytic property of DNA bases. One of the primary advantages of this technique is the rapid determination of mixing bases, even in the absence of enzymes and bisulfite. The analysis of DNA based on the chemical decomposition of oxidation is also possible. In this approach, 5-methylcytosine is separated [16]. At the beginning of this era, chemical oxidation decomposition–based methods have been discovered. In this method, 5-methylcytosine is isolated from cytosine using OsO_4_ and detects light-sensitive oxidation in the given sample. This mechanism influences the C5 and C6 bonds of methylcytosine, followed by the breakage of the bond using the piperidine method. The decomposition of DNA is then analyzed by electrophoresis of polyacrylamide gel, and methylation level is detected for the given test sample [4].

## 2. Role of DNA Methylation in Skin Disorders

DNA methylation is pivotal in carcinogenesis, influencing gene silence, chromatin remodeling, and oncogene activation in skin malignancies, including melanoma and squamous cell carcinoma. A research investigated m6A methylation alterations in skin cancer, illustrating the role of abnormal RNA methylation patterns in tumor growth and resistance to therapy. The research emphasized epigenetic targets including METTL3 and ALKBH5, which modulate m6A-dependent carcinogenic pathways. The findings indicate that methylation-based biomarkers and tailored medicines may be included into precision medicine strategies for skin cancer treatment.

### 2.1. Atopic Dermatitis (AD)

In AD, epidermal barrier dysfunction and immune deregulation are confirmed. Apart from this, gene regulation also plays a crucial role in disease progression. Different gene methylation profiling is evidence of disease progression via methylation alterations.

Due to lower DNMT1 mRNA levels in PBMCs of AD patients with high blood IgE levels, it has been suggested that DNMT1 inhibition and subsequent hypomethylation may play a role in this subset of AD patients. However, there has been limited research on the dysregulation of DNA methylation in AD [17]. In one study by Liang et al., it was found that monocytes from 10 AD patients showed global hypomethylation compared to healthy controls. They also discovered that the promoter of the Fc epsilon receptor 1 gamma (FCER1G), which correlated with gene expression, was hypomethylated in AD patients [18]. Additionally, when control monocytes were treated with the DNMT inhibitor azacytidine, hypomethylation occurred, leading to increased FcRI transcription and surface expression. In a related study, the role of thymic stromal lymphopoietin (TSLP) in disease progression was explored. The researchers observed that azacytidine treatment of KCs caused changes in skin lesions, including hypomethylation of the TSLP promoter, as well as an increase in TSLP mRNA and protein expression [19].

**Figure 2:**
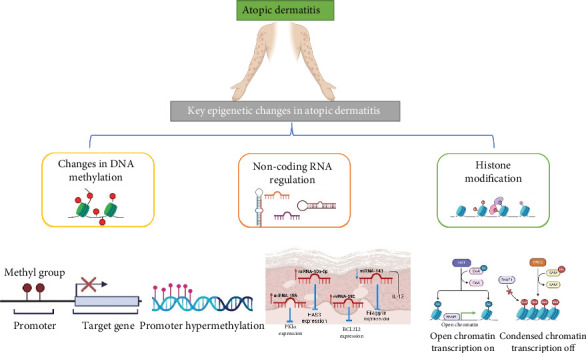
Atopic dermatitis inflammatory pathways: understanding epicenity.

Another scientist compared regular control patients' DNA methylation patterns (Figure 2) with diseased patients and concluded that DNA methylation changes were found in whole blood for the genes involved in nucleotide-binding oligomerization and pyrin domain–containing receptors (NLRPs), which are known to alter innate immunity via inducing NF-*κ*B activation. In addition to these multifarious investigations, DNA methylation at diverse CpG sites indicates amplified SIRL-1 expression in monocytes.

When tested, the scientists determined the methylation level in genes involved in disease progression, such as S100A2, S100A7, S100A8, S100A9, and S100A15. Among these genes, methylation changes were only found for S100A5, which was hypermethylated, as shown in Table 1. In KCs, the expressions of *KRT6A* and *KRT6B* and, in innate immune cells, the level of *OAS1*, *OAS2*, and *OAS3* escalated. OAS2 shows decreased methylation [37].

Another study claimed that approximately 25 genes were hypermethylated and 10 were hypomethylated in CD4+ CLA+ T cells presented in AD patients compared to control [38]. Demethylations of the VSTM1 promoter gene can also lead to AD as it influences the expression of SIRL-1 in monocytes. Production of reactive oxygen species is limited by the SIRL-1, which is related to the pathogenesis of AD [22]. In addition, another finding reported differential methylation at CpG sites of the KIF3 A gene, which gives an idea of the impact of DNA methylation on AD development [39].

### 2.2. Eczema

**Figure 3:**
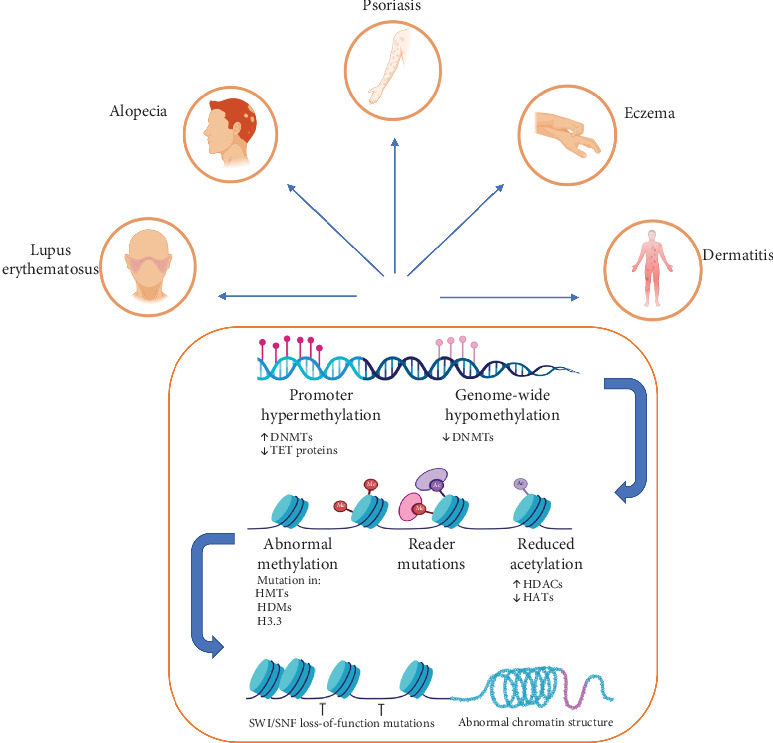
DNA methylation in skin disorders.

Eczema herpeticum (EH) is a worsening state of AD in which HSV-1 exposure is a prime factor for disease progression (Figure 3). Apart from this, ADEH+ patients represent the severity of the disease because the action of interferon is attenuated and the serum level of IgE is elevated. The exact mechanism is still unclear. Many studies have suggested the role of DNA methylation in disease development that participated in autoimmune disorders [23].

The filaggrin gene (FLG) protects the skin by creating a barrier to reduce water loss from the skin. The expression of FLG was decreased, leading to an increase in eczema, as shown in Table 1. CpG (cg18593727) and CpGs in IL4 (cg23943829) and in IL13 (cg04303330) were differently methylated when tested in the laboratory in ADEH+ patients.

### 2.3. Androgenetic Alopecia (AGA)

AGA is the baldness due to the androgen receptor gene due to an excessive response to androgens. Androgen receptor activation causes the growth phase or anagen phase of the usual hair growth cycle to be shortened. In cases of AGA, excessive activation leads to follicular shrinkage through a gradually shortened anagen phase, resulting in thinner and shorter hair follicles that may not even eventually reach the epidermis [40].

One study performed by Martinez-Jacobo determined the presence of higher AR methylation levels in occipital follicles. This may lead to protection of hair follicles against hair loss and miniaturization [24]. DNMT1 is crucial in establishing androgenic alopecia as it is required to regulate methylation patterns. Reduced expression of DNAMT1 can trigger baldness, which suggests a gene role in the progression of androgenic alopecia [24].

### 2.4. Systemic Lupus Erythematosus (SLE)

T and B cell overstimulation can lead to SLE. SLE is an autoimmune disease that causes organ damage. Along with environmental factors, genetics also play a role, but the molecular mechanisms are still unclear [28].

Most recently, altered DNA methylation profiles of various genes have been addressed. The gene for killer cell immunoglobulin-like receptor 2DL4 (KIR2DL4) was found to be demethylated when tested [41]. Apart from this, increased expression of serine/threonine protein phosphatase 2A (PP2A) is found in T cells from SLE patients where this reduction in methylation of sites containing CpG in the proximal promoter of the PP2A gene is the factor that causes the elevation in gene expression [25]. After comparison with normal control patients, it was annotated that women with SLE, as well as those with rheumatoid arthritis, have DNA methylation in the proximal promoter of the estrogen receptor 1 (ESR1) gene, which codes for the extensively expressed estrogen receptor *α* [17, 42].

Proinflammatory effector cytokines and IL-2 are not expressed by T cells from SLE patients due to increased DNA methylation of IL2 regulatory domains. In contrast to prior research, T cells from SLE patients showed enhanced DNA methylation of Foxp3 gene regulatory regions [27, 28, 43]. SLE patients' site-specific DNA methylation in the CD8 gene cluster leads to decreased activity of the CD8 coreceptor, as shown in Table 1. This alteration will cause an increase in DN T cells responsible for revealing proinflammatory effector cytokines containing IFN-*γ*, IL-17F, IL-12, IL-18, and others.

Overall, these investigations contribute to the role of modulation in DNA methylation levels in T cell subset commitment and lineage-specific cytokine expression. Moving further towards lymphocytes, global methylation is decreased in SLE patients [25].

### 2.5. Psoriasis

Psoriasis affects 125 million individuals worldwide. Hyperproliferative KCs and invading immune cells are characteristics of the chronic and recurring and inflammatory skin condition psoriasis. Even though the exact pathogenesis is still unclear, many studies have introduced the role of genetic factors in psoriasis [33]. A research confirmed that the level of DNA methylation in skin lesions is elevated compared to that of normal skin.

DNA methylation alterations are located in the promoter and first gene exon, which regulate the transcription process [30]. Compared to DNase I hypersensitivity sites, non-DNase I hypersensitive sites had a higher methylation level [30]. There are differences in the methylation of CG05590156, which is present in the TRIO gene that codes for the GEF (guanine nucleotide exchange factor) of the RHOA and RAC1 GTPases and serves a number of biological functions. Additionally, the psoriasis candidate genes CYP2S1 (CG19430423) and EIF2C2 (CG00288598) were shown to have varying amounts of methylation [30].

The methylation level also controls the gene expression of S100Aug, a gene responsible for cell proliferation and immune mechanism [44]. Furthermore, it was demonstrated that 12 other genes, including TRIM22, OAS2, MAN1C1, EIF2C2, ECE1, CYP2S1, C10orf99, and DLGAP4, had negative correlations with the degree of methylation, whereas the expression of three genes, GDPD3, TRIM14, and CCND1, correlated favorably with the degree of methylation [31]. More than 33% methylation difference has been observed in genes Wnt7B, NFATc1, CELSR2, and FZD7. In terms of methylation patterns of skin on different body areas, methylation in the abdomen and back is lower than in the limbs [45].

Patients with psoriasis have hypomethylated human leukocyte antigen (HLA)-DMA and G protein-coupled receptor 128 (GPR128) genes, but they have hypermethylated tenascin-XB (TNXB) and macrophage stimulating 1 receptor (MST1R) genes [32]. PBMCs contain dendritic cells, phagocytes, monocytes, lymphocytes, and a few other cell types. According to a study, psoriasis patients' PBMCs express more DNMT1 than those from the healthy control group [46]. Compared to healthy patients, individuals with psoriasis had hypermethylated CD4+ T cell chromosomes at high methylation levels. Other hypermethylated genes are PPAPDC3, TP73, and FANK1 [33].

### 2.6. Systemic Sclerosis (SSc)

SSc includes problems with internal organs, blood vessels, the digestive tract, and skin, which is tighter and more challenging. SSc is also known as scleroderma. The primary factors of disease development include early endothelial injury, an inflammatory infiltration, and a subsequent fibrotic response despite the complicated pathophysiology of scleroderma [47].

Ying Luo determined the level of DNA methylation in African American patients. He found that of 3.8 million CpG sites, approximately 1180 CpGs are differentially methylated in diseased patients. *DLX5*, a gene responsible for cell proliferation, was found to be hypermethylated in skin fibroblast [48]. *TMEM140* and *MCG12916* are also in a hypermethylated form in the same patients where *DLX5* and *TMEM140* showed expression, and *MCG12916* is downregulated in samples of the same patients. The findings of this study showed that, with the exception of CpG islands, where differentially methylated CpGs (DMCs) were hypermethylated, other regions displayed notable CpG hypomethylation [34]. Additionally, peripheral blood mononuclear cells from individuals with SSc demonstrated hypomethylation of the IFN regulatory factor 7 (IRF7) promoter [49].

Another study tested the methylation pattern in juvenile patients, and the results suggested a global reduction in methylation levels in CD4+ T cells. This further suggests that this may lead to reduced expression of DNMT1 [50]. Changes in methylation patterns were unique in both cases, where differentially methylated gene count is more in localized scleroderma (jLS) compared to juvenile-onset systemic sclerosis (jSSc) [51]. Regarding methylation level, most genes are hypermethylated in blood samples. *ARRB1* is hypomethylated in jLS patients, a gene belonging to the arrestin gene family [36]. TEAD3 is hypermethylated, a gene responsible for cell proliferation and contact activities [52]. The ITCH gene is also hypermethylated and is only seen in jLS patients. The function of this gene is to regulate immune function [53]. Differential methylation of ACTN1 and CAPN8 in jLS patients was also discovered [54]. Both jSSc and jLS patients had higher levels of DNA methylation than controls at CpG sites in the FGFR2 5⁣′-UTR, while jLS patients' levels exceeded those of jSSc patients' [28].

## 3. The Function of DNA Methylation–Related Enzymes and Inhibitors in Skin Disorders

DNA methylation is essential for epigenetic regulation, influencing gene expression and cellular differentiation. Dysregulation of DNA methylation in skin disorders contributes to inflammatory reactions, cancer, and compromised cellular function. DNMTs, such as DNMT1, DNMT3A, and DNMT3B, are the principal enzymes tasked with the establishment and maintenance of methylation patterns within the genome. Aberrant DNA methylation, typically characterized by hypermethylation of tumor suppressor genes or hypomethylation of oncogenes, has been associated with illnesses including psoriasis, AD, and skin cancer. The suppression of DNMT activity has become a promising therapeutic approach to influence epigenetic regulation and alleviate disease pathogenesis.

Recent research has yielded compelling evidence endorsing DNMT inhibitors as prospective treatment agents. Research revealed that 5-aza-2⁣′-deoxycytidine (5-Aza-dC), a DNMT inhibitor, greatly reduces psoriasiform inflammation caused by di (2-ethylhexyl) phthalate (DEHP). This work demonstrated that the suppression of DNMT activity resulted in diminished global DNA methylation levels in both dermal and peripheral immune compartments, correlating with reduced generation of inflammatory cytokines and immune cell infiltration in the afflicted skin areas [55]. Likewise, research reported the discovered dihydromyricetin, a natural DNMT inhibitor with antiaging and anti-inflammatory effects in the human skin. The research demonstrated that dihydromyricetin counteracts age-related hypermethylation in skin fibroblasts, resulting in enhanced collagen expression and diminished oxidative stress, indicating its prospective application in antiaging and dermatological treatments [56]. The results of these research underscore the possible therapeutic uses of DNMT-targeting inhibitors in skin-related inflammatory and degenerative disorders. In light of the increasing interest in epigenetic therapeutics, the review should enhance its discourse on DNMT inhibitors and their function in regulating DNA methylation for dermatological disorders, along with the special issue's emphasis.

## 4. Molecular Docking Investigations and Computational Methods in DNA Methylation Suppression

The progress of computational techniques has greatly aided in the development and optimization of enzyme inhibitors, particularly those aimed at DNMTs. Molecular docking and molecular dynamics simulations enable researchers to forecast binding affinities, structural interactions, and pharmacokinetics of prospective inhibitors, thereby expediting drug discovery and development. A group of researchers performed a molecular docking analysis of coumarin derivatives, assessing their inhibitory effects on tyrosinase and their antioxidative properties in skin disorders. The research indicated that certain coumarin derivatives possess a high binding affinity for tyrosinase, effectively inhibiting melanin production in vitro [57]. The work concentrated on tyrosinase inhibition; however, the technology can be adapted to discover and enhance DNMT inhibitors for epigenetic modulation in dermatological conditions.

The integration of computational modeling tools, including docking simulations, molecular dynamics analyses, and machine learning–driven drug design, will augment our comprehension of DNMT inhibitory processes. Subsequent research ought to investigate AI-driven methodologies for drug discovery aimed at developing innovative epigenetic regulators for the treatment of skin disorders, complementing the special issue's emphasis on enzyme inhibition and molecular modeling. The function of microRNAs (miRNAs) in DNA methylation and dermatological disorder miRNAs are crucial posttranscriptional regulators of gene expression, and their activity is closely associated with DNA methylation pathways. Dysregulated miRNA expression contributes to immunological dysregulation, KC impairment, and chronic inflammation in dermatological conditions such as psoriasis, AD, and melanoma. Research established that DNA methylation of microRNA-365-1 facilitates apoptosis in hair follicle stem cells by targeting DAP3, a mitochondrial-associated protein implicated in cell survival pathways [55, 58]. The research demonstrated that hypermethylation of miR-365-1 causes its inhibition, subsequently activating DAP3, which leads to death of hair follicle stem cells and hair loss [55, 59]. The findings indicate that the epigenetic regulation of miRNAs is essential for the survival and regeneration of skin cells, potentially impacting hair loss disorders. Research study examined the influence of miRNAs on inflammatory and immunological pathways in skin disorders, emphasizing the effects of dysregulated miRNA expression on KC differentiation and cytokine generation. miR-146a and miR-21 are significantly elevated in psoriasis and AD, resulting in enhanced proinflammatory cytokine secretion and immune cell infiltration in the afflicted dermal tissues [60]. A research study conducted a comprehensive investigation of epigenetic regulators in the inflammatory pathways of AD, associating DNA methylation changes with immune dysregulation and skin barrier impairment [61]. These findings underscore the necessity of targeting DNA methylation–mediated miRNA suppression as a prospective treatment approach for inflammatory skin disorders.

## 5. Conclusion

DNA methylation is pivotal in the advancement of numerous skin disorders by modulating gene expression without modifying the genetic sequence. Recent progress in epigenetic research has unveiled innovative therapeutic approaches focused on inhibiting DNA methylation–related enzymes, specifically DNMTs. Inhibiting DNMT activity has demonstrated encouraging outcomes in alleviating inflammatory skin disorders including psoriasis and AD by decreasing global DNA methylation levels and curtailing inflammatory cytokine production. Moreover, natural substances exhibiting DNMT inhibitory characteristics have shown promise for antiaging and dermatological treatments.

Molecular docking research and computational modeling have enhanced the identification and optimization of DNMT inhibitors, providing significant insights into enzyme–ligand interactions. These methodologies establish a basis for AI-facilitated drug discovery, advancing the formulation of tailored epigenetic treatments for dermatological conditions. In addition to DNMT inhibition, miRNA-mediated regulation of DNA methylation has become a crucial element in skin pathology. The dysregulation of particular miRNAs affects inflammatory pathways, skin cell viability, and immunological responses, highlighting the necessity to investigate DNA methylation–targeted treatment strategies for immune-mediated skin conditions. In the era of skin cancer, anomalous DNA methylation patterns facilitate tumor advancement and therapeutic resistance. Methylation-targeted treatments, such as DNMT inhibitors and histone deacetylase inhibitors, possess potential in precision medicine for the treatment of melanoma and other cancers. Progress in epigenetic research consistently unveils novel opportunities for formulating revolutionary therapeutic strategies that modulate DNA methylation. Comprehending and addressing these pathways would facilitate the development of more efficacious therapeutic options for controlling skin diseases, providing optimism for enhanced patient outcomes.

## Figures and Tables

**Table 1:** Clinical evidence suggesting the role of DNA methylation in skin diseases.

**Sr. no.**	**Disease name**	**Methylated gene**	**Method**	**Sample**	**Status**	**Reference**
1	Atopic dermatitis	Monocytes	Infinium 27K methylation assay	Monocytes	Hypomethylated	[20]
		Fc epsilon receptor 1 gamma (FCERIG)	Infinium 27K methylation assay	Monocytes	Hypomethylated	[20]
		Thymic stromal lymphopoietin (TSLP)	Bisulfite sequencing	Skin samples	Hypomethylated	[21]
		S100A5	Infinium 27K methylation assay	Whole blood samples	Hypermethylated	[20]
		*OAS2*	Infinium 27K methylation assay	Whole blood samples	Hypomethylated	[20]
		CFLAR, GPR55, MMP7, LOC283487, SH2D2A, and ERP27	Infinium 27K methylation assay	Whole blood samples	Hypomethylated	[20]
		LRRC8C, S100A5, and EBP49	Infinium 27K methylation assay	Whole blood samples	Hypermethylated	[20]
		IL23A	Infinium 27K methylation assay	Whole blood samples	Hypomethylated	[20]
		KRT6A	Infinium 27K methylation assay	Whole blood samples	Hypomethylation	[20]
		VSTM1 promoter gene	Bisulfite sequencing	Human blood samples	Demethylated	[22]

2	Eczema	IL4 (cg23943829)	Genome-wide tests	Blood	Differential methylation	[23]
		IL13 (cg04303330),	Genome-wide tests	Blood	Differential methylation	[23]

3	Androgenetic alopecia	Androgen receptor	Polymerase chain reaction	Hair follicles	Hypermethylated	[24]

4	Systemic lupus erythematosus (SLE)	Killer cell immunoglobulin-like receptor 2DL4 (*KIR2DL4*)	EZ DNA methylation kit method	Peripheral blood mononuclear cells (PBMC)	Demethylated	[25]
		Proximal promoter of the PP2A gene	Methyl flash methylated DNA quantification kit	Primary T cells	Hypomethylated	[26]
		IL2 regulatory regions	The methylated CpG-DNA immunoprecipitation assay	CD4+ T cells	Hypermethylated	[27]
		Proinflammatory effector cytokines	EZ DNA methylation kit method	Cytokines	Hypermethylated	[28]
		Foxp3 gene	Bisulfite sequencing	CD4+ T cells from patients	Hypermethylation	[29]
		Lymphocytes	Bisulfite sequencing	Lymphocytes	Hypomethylated	[29]

5	Psoriasis	Skin lesions	Stage I epigenome-wide association analysis	Skin and peripheral blood mononuclear cell samples	Hypermethylation	[30]
		Non-DNase I hypersensitive sites	Stage I epigenome-wide association analysis	Skin and peripheral blood mononuclear cell samples	Hypermethylation	[30]
		CYP2S1 (CG19430423)	Stage I epigenome-wide association analysis	Skin and peripheral blood mononuclear cell samples	Differentially methylated	[30]
		EIF2C2 (CG00288598)	Infinium HumanMethylation27 Beadchip	Skin	Differentially methylated	[31]
		CYP2S1, ECE1, EIF2C2, MAN1C1, DLGAP4, OAS2, LGALS3BP	Infinium HumanMethylation27 Beadchip	Skin	Decreased methylation level	[31]
		KYNU, IL1B, TRIM22, and PHYHIP	Infinium HumanMethylation27 Beadchip	Skin	Decreased methylation level	[31]
		GPR128 (G protein-coupled receptor 128) and HLA-DMA (human leukocyte antigen-DMA)	Genome-wide DNA methylation profiling	Skin	Hypomethylated	[32]
		Macrophage stimulating 1 receptor (MST1R) and tenascin-XB (TNXB)	Genome-wide DNA methylation profiling	Skin	Hypermethylated	[32]
		CD4+ T cell chromosomes	Western blot assay	Human keratinocytes and fibroblast-like synoviocytes	Hypermethylated	[33]
		PPAPDC3, TP73, and FANK1	Western blot assay	Human keratinocytes and fibroblast-like synoviocytes	Hypermethylated	[33]

6	Systemic sclerosis	*DLX5* a gene	Bismarck v0.16.3	Forearm skin	Hypermethylated	[34]
		*TMEM140*	Bismarck v0.16.3	Forearm skin	Hypermethylated	[34]
		*MCG1291*	Bismarck v0.16.3	Forearm skin	Hypermethylated	[34]
		IFN regulatory factor 7 (IRF7) promoters	EZ DNA methylation kit	Whole blood samples	Hypomethylated	[35]
		*ARRB1*	EZ DNA methylation kit	Whole blood samples	Hypomethylated	[36]
		*TEAD3*	EZ DNA methylation kit	Whole blood samples	Hypermethylated	[35]
		5⁣′-UTR of *FGFR2*	EZ DNA methylation kit	Whole blood samples	Hypermethylated	[35]

## Data Availability

Data sharing is not applicable to this article as no datasets were generated or analyzed during the current study.

## References

[1] Arda O., Göksügür N., Tüzün Y. (2014). Basic histological structure and functions of facial skin. *Clinics in Dermatology*.

[2] Gerlach E., Dreisbach R. H., Deuticke B. (1965). Paper chromatographic separation of nucleotides, nucleosides, purines, and pyrimidines. *Journal of Chromatography A*.

[3] Moore L. D., Le T., Fan G. (2013). DNA methylation and its basic function. *Neuropsychopharmacology*.

[4] Khodadadi E., Fahmideh L., Khodadadi E. (2021). Current advances in DNA methylation analysis methods. *BioMed Research International*.

[5] Zhang L., Xu Y. Z., Xiao X. F. (2015). Development of techniques for DNA-methylation analysis. *TrAC Trends in Analytical Chemistry*.

[6] Yong W. S., Hsu F. M., Chen P. Y. (2016). Profiling genome-wide DNA methylation. *Epigenetics & Chromatin*.

[7] Candiloro I. L., Mikeska T., Dobrovic A. (2011). Assessing combined methylation–sensitive high resolution melting and pyrosequencing for the analysis of heterogeneous DNA methylation. *Epigenetics*.

[8] Meissner A., Gnirke A., Bell G. W., Ramsahoye B., Lander E. S., Jaenisch R. (2005). Reduced representation bisulfite sequencing for comparative high-resolution DNA methylation analysis. *Nucleic Acids Research*.

[9] Yokoyama S., Kitamoto S., Yamada N. (2012). The application of methylation specific electrophoresis (MSE) to DNA methylation analysis of the 5′ CpG island of mucin in cancer cells. *BMC Cancer*.

[10] Hernández H. G., Tse M. Y., Pang S. C., Arboleda H., Forero D. A. (2013). Optimizing methodologies for PCR-based DNA methylation analysis. *BioTechniques*.

[11] Hussmann D., Hansen L. L. (2018). Methylation-sensitive high resolution melting (MS-HRM). *DNA Methylation Protocols*.

[12] Qin X., Xu J., Zhong Y. (2017). *Multidisciplinary management of liver metastases in colorectal cancer: Early diagnosis and treatment*.

[13] Luo X., Li Y., Zheng J., Qi H., Liang Z., Ning X. (2015). The determination of DNA methyltransferase activity by quenching of tris (2, 2′-bipyridine) ruthenium electrogenerated chemiluminescence with ferrocene. *Chemical Communications*.

[14] Tanaka K., Tainaka K., Kamei T., Okamoto A. (2007). Direct labeling of 5-methylcytosine and its applications. *Journal of the American Chemical Society*.

[15] Wang P., Wu H., Dai Z., Zou X. (2011). Simultaneous detection of guanine, adenine, thymine and cytosine at choline monolayer supported multiwalled carbon nanotubes film. *Biosensors and Bioelectronics*.

[16] Yamada H., Tanabe K., Nishimoto S. I. (2006). Sensitive discrimination between cytosine and 5-methylcytosine in DNA by a modified invader method. *Nucleic Acids Symposium Series*.

[17] Schmidt A. D., de Guzman Strong C. (2021). Current understanding of epigenetics in atopic dermatitis. *Experimental Dermatology*.

[18] Novak N., Bieber T., Leung D. Y. M. (2003). Immune mechanisms leading to atopic dermatitis. *Journal of Allergy and Clinical Immunology*.

[19] Ziyab A. H., Karmaus W., Holloway J. W., Zhang H., Ewart S., Arshad S. H. (2013). DNA methylation of the filaggrin gene adds to the risk of eczema associated with loss-of-function variants. *Journal of the European Academy of Dermatology and Venereology*.

[20] Rodríguez E., Baurecht H., Wahn A. F. (2014). An integrated epigenetic and transcriptomic analysis reveals distinct tissue-specific patterns of DNA methylation associated with atopic dermatitis. *Journal of Investigative Dermatology*.

[21] Luo Y., Zhou B., Zhao M., Tang J., Lu Q. (2014). Promoter demethylation contributes to TSLP overexpression in skin lesions of patients with atopic dermatitis. *Clinical and Experimental Dermatology*.

[22] Kumar D., Puan K. J., Andiappan A. K. (2017). A functional SNP associated with atopic dermatitis controls cell type-specific methylation of the *VSTM1* gene locus. *Genome Medicine*.

[23] Boorgula M. P., Taub M. A., Rafaels N. (2019). Replicated methylation changes associated with eczema herpeticum and allergic response. *Clinical Epigenetics*.

[24] Martinez-Jacobo L., Villarreal-Villarreal C. D., Ortiz-López R., Ocampo-Candiani J., Rojas-Martínez A. (2018). Genetic and molecular aspects of androgenetic alopecia. *Indian Journal of Dermatology, Venereology and Leprology*.

[25] Hedrich C. M., Tsokos G. C. (2011). Epigenetic mechanisms in systemic lupus erythematosus and other autoimmune diseases. *Trends in Molecular Medicine*.

[26] Sunahori K., Nagpal K., Hedrich C. M., Mizui M., Fitzgerald L. M., Tsokos G. C. (2013). The catalytic subunit of protein phosphatase 2A (PP2Ac) promotes DNA hypomethylation by suppressing the phosphorylated mitogen-activated protein kinase/extracellular signal-regulated kinase (ERK) kinase (MEK)/phosphorylated ERK/DNMT1 protein pathway in T-cells from controls and systemic lupus erythematosus patients. *Journal of Biological Chemistry*.

[27] Hedrich C. M., Rauen T., Tsokos G. C. (2011). cAMP-responsive element modulator (CREM) *α* protein signaling mediates epigenetic remodeling of the human interleukin-2 gene: implications in systemic lupus erythematosus. *Journal of Biological Chemistry*.

[28] Hedrich C. M., Mäbert K., Rauen T., Tsokos G. C. (2017). DNA methylation in systemic lupus erythematosus. *Epigenomics*.

[29] Wang Y. Y., Wang Q., Sun X. H. (2014). DNA hypermethylation of the forkhead box protein 3 (*FOXP3*) promoter in CD4^+^ T cells of patients with systemic sclerosis. *British Journal of Dermatology*.

[30] Zhou F., Wang W., Shen C. (2016). Epigenome-wide association analysis identified nine skin DNA methylation loci for psoriasis. *Journal of Investigative Dermatology*.

[31] Roberson E. D., Liu Y., Ryan C. (2012). A subset of methylated CpG sites differentiate psoriatic from normal skin. *Journal of Investigative Dermatology*.

[32] Chandra A., Senapati S., Roy S., Chatterjee G., Chatterjee R. (2018). Epigenome-wide DNA methylation regulates cardinal pathological features of psoriasis. *Clinical Epigenetics*.

[33] Luo Y., Qu K., Kuai L. (2021). Epigenetics in psoriasis: perspective of DNA methylation. *Molecular Genetics and Genomics*.

[34] Baker Frost D., da Silveira W., Hazard E. S. (2021). Differential DNA methylation landscape in skin fibroblasts from African Americans with systemic sclerosis. *Genes*.

[35] Coit P., Schollaert K. L., Mirizio E. M., Torok K. S., Sawalha A. H. (2021). DNA methylation patterns in juvenile systemic sclerosis and localized scleroderma. *Clinical Immunology*.

[36] Gurevich V. V., Gurevich E. V. (2019). GPCR signaling regulation: the role of GRKs and arrestins. *Frontiers in Pharmacology*.

[37] Han J., Park S. G., Bae J. B. (2012). The characteristics of genome-wide DNA methylation in naïve CD4^+^ T cells of patients with psoriasis or atopic dermatitis. *Biochemical and Biophysical Research Communications*.

[38] Bieber T. (2020). Interleukin-13: targeting an underestimated cytokine in atopic dermatitis. *Allergy*.

[39] Johansson E., Biagini Myers J. M., Martin L. J. (2017). KIF3A genetic variation is associated with pediatric asthma in the presence of eczema independent of allergic rhinitis. *Journal of Allergy and Clinical Immunology*.

[40] Ho C. H., Sood T., Zito P. M. (2024). Androgenetic alopecia. *StatPearls*.

[41] Liu Y., Kuick R., Hanash S., Richardson B. (2009). DNA methylation inhibition increases T cell KIR expression through effects on both promoter methylation and transcription factors. *Clinical Immunology*.

[42] Liu H. W., Lin H. L., Yen J. H. (2014). Demethylation within the proximal promoter region of human estrogen receptor alpha gene correlates with its enhanced expression: implications for female bias in lupus. *Molecular Immunology*.

[43] Lal G., Zhang N., Van Der Touw W. (2009). Epigenetic regulation of Foxp3 expression in regulatory T cells by DNA methylation. *The Journal of Immunology*.

[44] Semprini S., Capon F., Tacconelli A. (2002). Evidence for differential S100 gene over-expression in psoriatic patients from genetically heterogeneous pedigrees. *Human Genetics*.

[45] Tang L., Cheng Y., Zhu C. (2018). Integrative methylome and transcriptome analysis to dissect key biological pathways for psoriasis in Chinese Han population. *Journal of Dermatological Science*.

[46] Zhang P., Su Y., Chen H., Zhao M., Lu Q. (2010). Abnormal DNA methylation in skin lesions and PBMCs of patients with psoriasis vulgaris. *Journal of Dermatological Science*.

[47] Rosendahl A. H., Schönborn K., Krieg T. (2022). Pathophysiology of systemic sclerosis (scleroderma). *The Kaohsiung Journal of Medical Sciences*.

[48] Altorok N., Tsou P. S., Coit P., Khanna D., Sawalha A. H. (2015). Genome-wide DNA methylation analysis in dermal fibroblasts from patients with diffuse and limited systemic sclerosis reveals common and subset-specific DNA methylation aberrancies. *Annals of the Rheumatic Diseases*.

[49] Rezaei R., Mahmoudi M., Gharibdoost F. (2017). IRF 7 gene expression profile and methylation of its promoter region in patients with systemic sclerosis. *International Journal of Rheumatic Diseases*.

[50] Zhou Y., Yuan J., Pan Y. (2009). T cell CD40LG gene expression and the production of IgG by autologous B cells in systemic lupus erythematosus. *Clinical Immunology*.

[51] Ramos P. S., Zimmerman K. D., Haddad S., Langefeld C. D., Medsger T. A., Feghali-Bostwick C. A. (2019). Integrative analysis of DNA methylation in discordant twins unveils distinct architectures of systemic sclerosis subsets. *Clinical Epigenetics*.

[52] Huh H. D., Kim D. H., Jeong H. S., Park H. W. (2019). Regulation of TEAD transcription factors in cancer biology. *Cells*.

[53] Melino G., Gallagher E., Aqeilan R. I. (2008). Itch: a HECT-type E3 ligase regulating immunity, skin and cancer. *Cell Death & Differentiation*.

[54] Ji J., Su L., Liu Z. (2016). Critical role of calpain in inflammation. *Biomedical Reports*.

[55] Liu X., Xi R., Du X. (2024). DNA methylation of microRNA-365-1 induces apoptosis of hair follicle stem cells by targeting DAP3. *Non-coding RNA Research*.

[56] Cai M., Li X., Luan X., Zhao P., Sun Q. (2024). Exploring m6A methylation in skin cancer: insights into molecular mechanisms and treatment. *Cellular Signalling*.

[57] Alfardan A. S., Nadeem A., Ahmad S. F. (2024). DNMT inhibitor, 5-aza-2′-deoxycytidine mitigates di (2-ethylhexyl) phthalate-induced aggravation of psoriasiform inflammation in mice via reduction in global DNA methylation in dermal and peripheral compartments. *International Immunopharmacology*.

[58] Chandarajoti K., Kara J., Suwanhom P. (2024). Synthesis and evaluation of coumarin derivatives on antioxidative, tyrosinase inhibitory activities, melanogenesis, and in silico investigations. *Scientific Reports*.

[59] Falckenhayn C., Bienkowska A., Söhle J. (2024). Identification of dihydromyricetin as a natural DNA methylation inhibitor with rejuvenating activity in human skin. *Frontiers in Aging*.

[60] Singhvi G., Manchanda P., Krishna Rapalli V., Kumar Dubey S., Gupta G., Dua K. (2018). MicroRNAs as biological regulators in skin disorders. *Biomedicine & Pharmacotherapy*.

[61] da Silva Duarte A. J., Sanabani S. S. (2024). Deciphering epigenetic regulations in the inflammatory pathways of atopic dermatitis. *Life Sciences*.

